# Metabolomic and transcriptomic analyses of yellow-flowered crocuses to infer alternative sources of saffron metabolites

**DOI:** 10.1186/s12870-024-05036-1

**Published:** 2024-05-07

**Authors:** Zahra Nemati, Seyyedeh-Sanam Kazemi-Shahandashti, Adriana Garibay-Hernández, Hans-Peter Mock, Maximilian H.-W. Schmidt, Björn Usadel, Frank R. Blattner

**Affiliations:** 1 Leibniz Institute of Plant Genetics and Crop Plant Research (IPK), Gatersleben, Germany; 2 Institute of Bio- and Geosciences (IBG-4: Bioinformatics), Bioeconomy Science Center (BioSC) , CEPLAS, Forschungszentrum Jülich GmbH, Jülich, Germany; 3 Institute for Biological Data Science, Faculty of Mathematics and Natural Sciences, Cluster of Excellence on Plant Sciences (CEPLAS), Heinrich Heine University Düsseldorf, Düsseldorf, Germany; 4 Institute of Grapevine Breeding, Geisenheim University, Geisenheim, Germany; 5 Present address: Institute of Medical Microbiology and hospital hygiene, Universitätsklinikum Frankfurt, Frankfurt am Main, Germany; 6 Molecular Biotechnology and Systems Biology, Technische Universität Kaiserslautern, Kaiserslautern, Germany

**Keywords:** Saffron-like compounds, Yellow-tepal crocuses, Metabolomics, Transcriptomics, Crocins, Flavonoids, Alternative sources

## Abstract

**Background:**

The increasing demand for saffron metabolites in various commercial industries, including medicine, food, cosmetics, and dyeing, is driven by the discovery of their diverse applications. Saffron, derived from *Crocus sativus* stigmas, is the most expensive spice, and there is a need to explore additional sources to meet global consumption demands. In this study, we focused on yellow-flowering crocuses and examined their tepals to identify saffron-like compounds.

**Results:**

Through metabolomic and transcriptomic approaches, our investigation provides valuable insights into the biosynthesis of compounds in yellow-tepal crocuses that are similar to those found in saffron. The results of our study support the potential use of yellow-tepal crocuses as a source of various crocins (crocetin glycosylated derivatives) and flavonoids.

**Conclusions:**

Our findings suggest that yellow-tepal crocuses have the potential to serve as a viable excessive source of some saffron metabolites. The identification of crocins and flavonoids in these crocuses highlights their suitability for meeting the demands of various industries that utilize saffron compounds. Further exploration and utilization of yellow-tepal crocuses could contribute to addressing the growing global demand for saffron-related products.

**Supplementary Information:**

The online version contains supplementary material available at 10.1186/s12870-024-05036-1.

## Highlights

• This study uncovers yellow-tepal crocuses as sources of saffron-like compounds, meeting industry demand. Metabolomic and transcriptomic research reveals insights into crocins and flavonoids biosynthesis, offering a promising production avenue.

**Supplementary Information:**

The online version contains supplementary material available at 10.1186/s12870-024-05036-1.

## Introduction

 The spice saffron consists of vivid crimson threads which are the dried stigmas of *Crocus sativus*. To obtain one kilogram of saffron 150,000-160,000 flowers are required. Literally, the small amount of saffron obtained per plant, along with its manual harvesting, leads to saffron being the most expensive spice in the world. From culinary uses and art crafts to medicine and health, the value of saffron has risen over the course of time. While saffron fulfills different ranges of commercial demands these days, its application is not limited to a spice or colorant [1]. It has a longstanding reputation in traditional medicine and researchers found biomedical and pharmacological properties of its metabolites. Saffron chemical composition is characterized as being rich in apocarotenoids and comprising kaempferol-derivatives as its sole flavonoid species. The apocarotenoids mainly consist of crocetin and its glucosyl esters, also named crocins (which occur as *trans-* and *cis-* isomers), 2,6,6-trimethyl-4-hydroxy-1-carboxyaldehyde-1-cyclohexene (HTCC), picrocrocin (which is glucosylated HTCC), and safranal, which is a product of picrocrocin catabolism [2]. Crocins and safranal are mainly responsible for saffron’s color and its unique aroma, respectively [3]. These metabolites also exert pharmacological effects on the prevention and/or improvement of various diseases related to the gastrointestinal, cardiovascular, endocrine, nervous, and immune systems [4].

Belonging to plant apocarotenoids [5], crocins are found in crocin-producing plants such as crocuses, *Buddleja davidii*, and *Gardenia jasminoides* [6]. The interaction among general isoprenoid pathways (MEP: methylerythritol phosphate and MVA: mevalonate pathways), carotenoid, and apocarotenoid biosynthetic pathways [7], results in the presence of a wide variety of crocins in crocin-producing plants. In case of saffron stigmas, these include crocin-I (*t*-C-4: *trans*-crocetin di(β-D-gentibiosyl) ester), with the highest abundance of more than 70% of all crocins, crocin-II (*t*-C-3: *trans*-crocetin (β-D-gentibiosyl) (β-D-glucosyl) ester), crocin-III (*t-*C-2: *trans*-crocetin (β-D-gentibiosyl) ester), crocin-IV (*t-*C-2: *trans*-crocetin di(β-D-glucosyl) ester), and crocin-V (*t-*C-1: *trans*-crocetin (β-D-glucosyl) ester) [8, 9]. These five forms differ regarding the position and number of glucosyl moieties attached to the crocetin aglycone backbone [10]. In the general isoprenoid pathways, the precursors of carotenoids are generated via MEP and MVA, followed by the activity of enzymes from the upstream carotenoid biosynthetic pathway such as phytoene synthase (PSY), lycopene β-cyclase (LCYB), and β-carotene hydrolase (BHY). The next stages of saffron apocarotenoid synthesis are accomplished by three key enzymes including the carotenoid cleavage dioxygenase (CCD2), aldehyde dehydrogenase (ALDH), as well as crocetin and HTCC glucosyltransferases (UGTs) [11]. The presence of the respective genes for these enzymes and their transcription activity can provide additional insights in the presence or potential presence of certain metabolic pathways [12–14].

Being also commercially valuable compounds, kaempferol and its glycosylated derivatives were also identified in crocuses. In fact, kaempferol and its derivatives comprise 90% of the total flavonoid content in tepals of *C. sativus* [15, 16]. Quercetin glycosides are the second most abundant flavonoid class in crocus tepals, comprising 5–10% of the flavonoid compounds, whereas other components like dihydrokaempferol glycosides and naringenin are observed in negligible amounts [15]. At the initial stage of the flavonoid biosynthetic pathway, phenylalanine is converted into *p-*coumaryl-CoA by the sequential activity of phenylalanine ammonia lyase, cinnamic acid 4-hydrolase, and *p*-coumaric acid: CoA ligase. The chalcone synthase catalyzes the first committed step into flavonoid biosynthesis through the condensation of *p-*coumaroyl-CoA with malonyl-CoA, resulting in the formation of naringenin chalcone, which the chalcone isomerase transforms into naringenin that is further metabolized into multiple flavonoids in a plant lineage-specific manner. Flavonoids are extensively decorated by diverse chemical reactions, including the glycosylation of flavonoid aglycones that is catalyzed by glycosyltransferases [17–19].

Regarding the growing demand for saffron, tepals of *C. sativus*, which are the major by-products of saffron production, could be an additional source of saffron-like specialized metabolites in addition to the plant’s stigmas [20]. The presence of crocin as well as kaempferol derivatives in tepals of C. sativus was confirmed through multiple analytical approaches such as liquid chromatography coupled to diode array detection (HPLC-DAD) and infrared (IR) spectroscopy [21]. These studies identified at least six different crocins in *C. sativus* tepals and the remarkable amount of kaempferol derivatives of 126 mg g^-1^ dry weight [21]. Owing to these metabolites’ presence in saffron tepals, they have been suggested as an alternative or supplementary medicine against some diseases [22] and effectively applied as natural pigments in natural and synthetic substrates [23].

*Crocus sativus* tepals are not the only extra source of these metabolites. There are about 250 accepted species within the genus *Crocus* (Iridaceae) occurring from the Mediterranean to western China [24–28]. They contain a broad spectrum of natural products including flavonoids, coumarins, additional phenolic glycosides, alkaloids, monoterpenoids, sesquiterpenoids, carotenoids, α-hydroxyacids, fatty alcohols, fatty aldehydes, fatty esters, fatty acids, and alkanes [29]. Within *Crocus*, compounds similar to those from saffron stigmas may occur in the tepals of different species. Being divided into spring- and autumn-flowering crocuses, the color of their flower’s ranges from white over yellow in spring, to purple and blue-lilac in autumn species [6]. In general, the presence of carotenoids and flavonoids in flowers leads to petal pigmentation in the range of yellow to orange [30]. While saffron’s purple tepals contain crocin and flavonoids, it is possible that the tepals of yellow crocuses also contain saffron-like metabolites due to their yellow color and thus, may be a potential source of these metabolites. Since saffron stigmas and petals are the sources of saffron main metabolites in autumn, yellow-tepal crocuses could increase the yield per year if they contain saffron-like metabolites.

In this study, we compared the semi-polar metabolite profiles of the four yellow-tepal *Crocus* taxa *C. chrysanthus*, *C. flavus* subsp. *dissectus*, *C. graveolens*, and *C. korolkowii* via LC-PDA analysis to investigate the presence of compounds similar to those in saffron stigmas. We also provide insights into the biosynthesis of crocins and kaempferol derivatives in yellow-tepal crocuses through their transcriptomic analysis with special focus on the active genes involved in carotenoid and flavonoid metabolism.

## Results

**Fig. 1:**
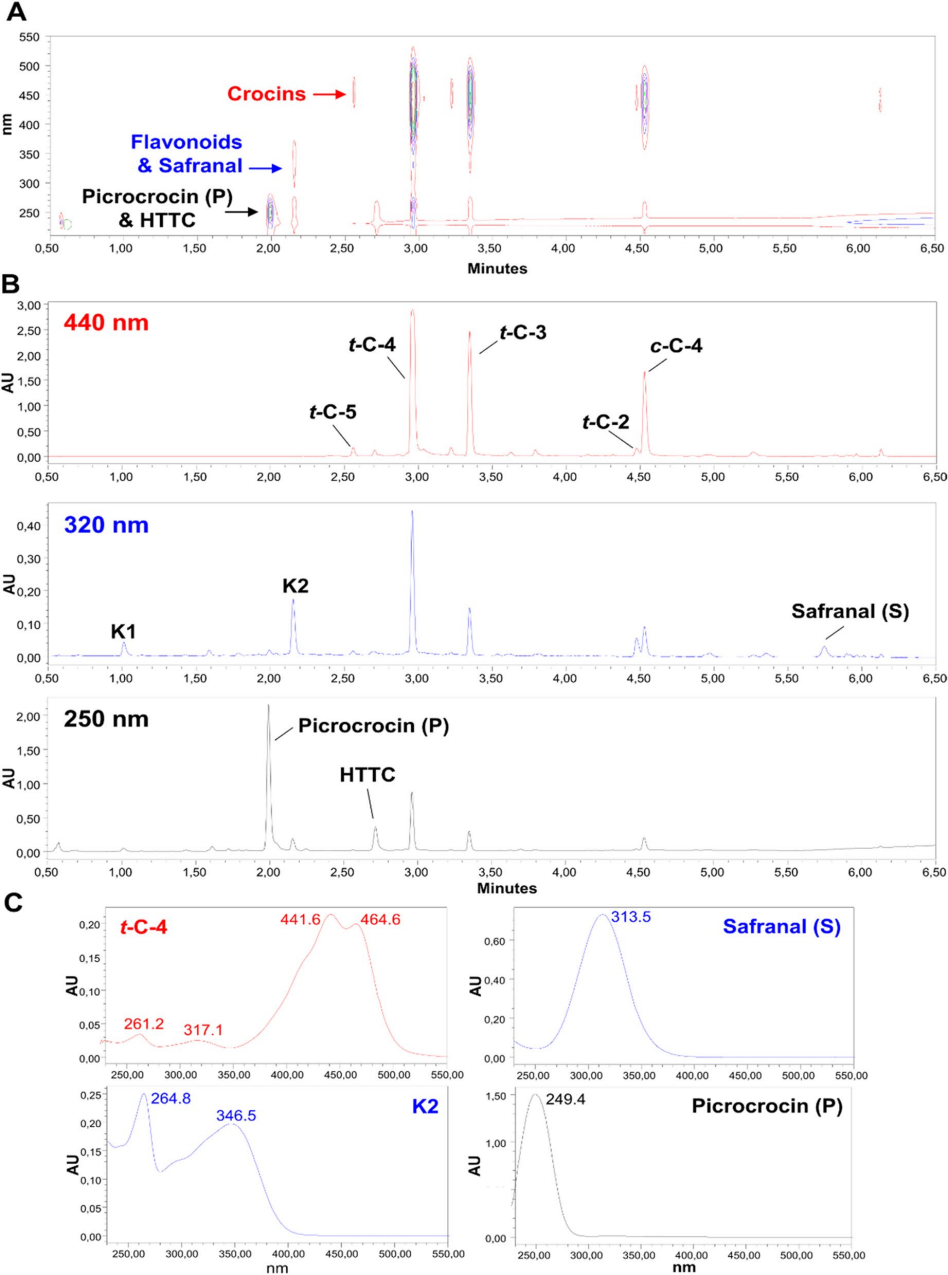
Major soluble semi-polar metabolites in *C. sativus* stigmas. **A** Representative LC-PDA isoplot chromatogram of semi-polar compounds detected within the range of 230–550 nm in extracts from *C. sativus* stigmas. Three major compound classes displaying maximum UV-Vis absorption values at specific wavelengths are indicated. **B** LC-PDA chromatograms extracted at 440, 320, and 250 nm, showing three major groups of semi-polar metabolites in *C. sativus* stigmas. **C** Representative UV-Vis absorption spectra for selected stigma metabolites

To investigate the presence of apocarotenoids and flavonoids similar to those of *C. sativus* stigmas in yellow-tepal crocus plants, the composition of *C. sativus* stigmas was initially assessed through LC-PDA-MS (Fig. 1).

We annotated a total of ten major compounds in *C. sativus* stigmas comprising both, apocarotenoids and flavonoids: (i) safranal, (ii) HTCC (picrocrocin precursor), (iii) picrocrocin, (iv) four *trans*-crocins including the well-known saffron crocin *trans*-crocetin-digentibiosyl ester (*t*-C-4), (v) one *cis*-crocin (*c-*C-4), and (vi) two major flavonoids, kaempferol 3*-O-*sophoroside 7*-O-*glucoside (K1) and kaempferol 3*-O-*sophoroside (K2). The most abundant crocins in the stigma samples were *t-*C-3, *t-*C-4, and *c-*C-4, while the crocins *t-*C-2 and *t-*C-5 were identified in lower abundance (Fig. 1).

**Fig. 2:**
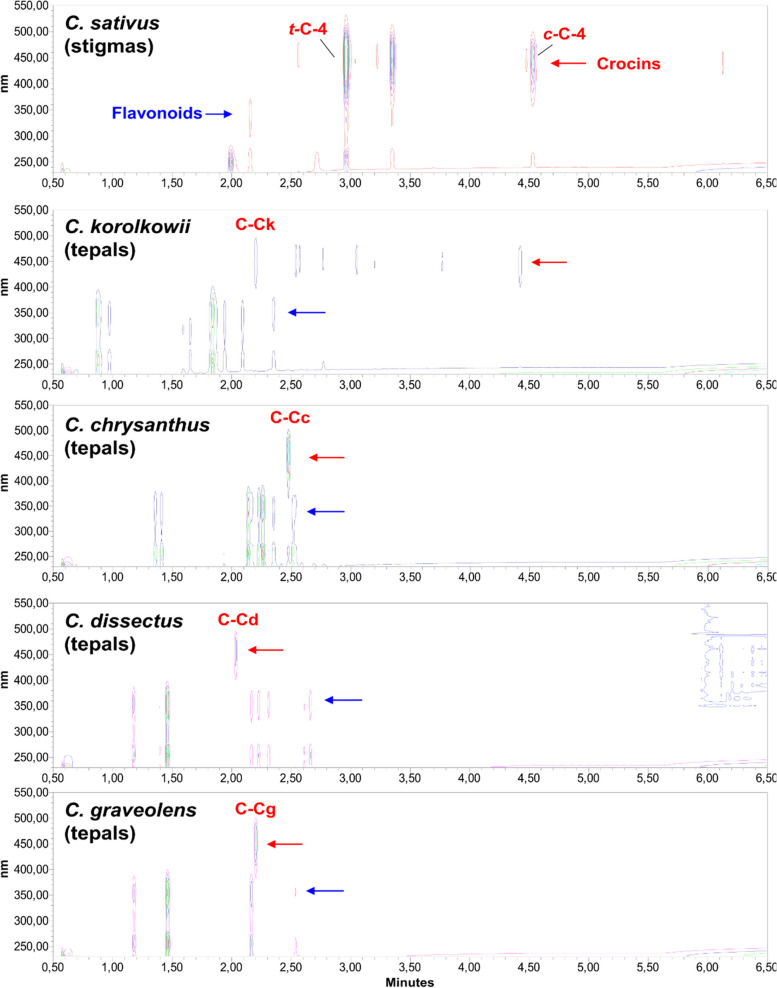
Major soluble semi-polar metabolites in yellow-tepal Crocus species compared to *C. sativus* stigmas. LC-PDA isoplot chromatograms of semi-polar compounds detected within the range of 230–550 nm in extracts from *C. sativus* stigmas and yellow tepals from different Crocus species. The UV-Vis absorption regions where crocins and flavonoids display their maximum absorbance are indicated with red and blue arrows, respectively. Specific crocins are labeled (red color); their UV-Vis absorption spectra are shown in Supplementary Fig. S1

**Fig. 3:**
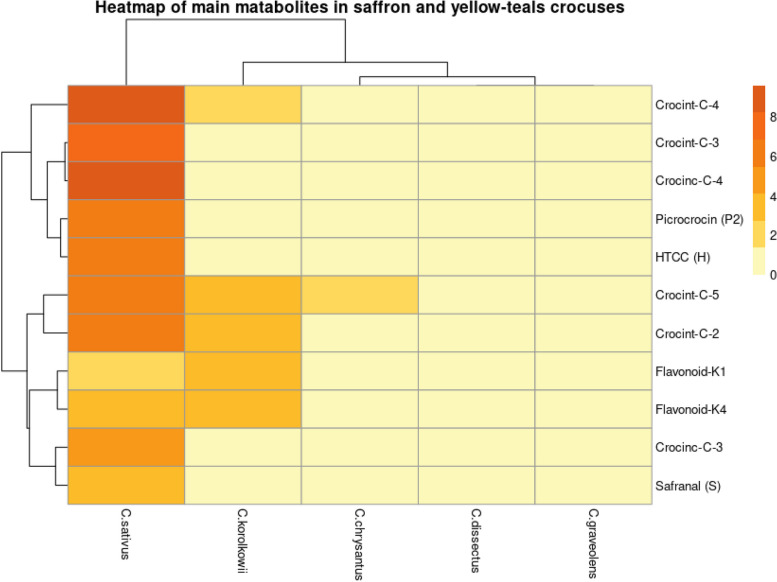
Heatmap of saffron-like metabolites. Metabolites (relative abundance) are represented on the right-hand side of the plot and the Crocus species are indicated in the bottom of the heatmap

After having established this baseline, we compared via LC-PDA the composition of the stigmas from *C. sativus* to that of yellow-tepals of *C. chrysanthus*, *C. flavus* subsp. *dissectus*, *C. graveolens*, and *C. korolkowii* to determine whether similar compounds can be found (Figs. 2 and 3). A heatmap shows the metabolite composition of *C. sativus* stigmas compared to that from tepals of yellow-tepal crocuses (Fig. 3, Table S1; tables and figures indicated by “S” are available as supplemental online materials attached to this article). We did not find the monoterpenoids HTCC, picrocrocin, and safranal in any of the yellow-tepal samples. In contrast, the yellow-tepals from *C. chrysanthus* and *C. korolkowii* shared the capability of synthesizing *trans-*crocins that are also found in saffron stigmas. The tepals from these two species displayed the *t*-C-5 crocin, whereas only *C. korolkowii* also showed *t*-C-4 and *t*-C-2 (Fig. 1), which are the major crocins found in saffron stigmas [14]. Interestingly, the yellow-tepals from all analyzed *Crocus* species displayed additional crocins in their profiles, as confirmed by their UV-Vis spectra (Fig. 2, S1, S2). These major crocins detected in the tepal samples did not correspond to the dominant crocins in *C. sativus* stigmas (Fig. S2).

The tepals displayed rich flavonoid profiles that clearly differ from those of saffron stigmas (Fig. 2, S3). Only *C. korolkowii* displayed the two kaempferol derivatives that we also found in the saffron stigmas, kaempferol 3-O-sophoroside and kaempferol 3-O-sophoroside 7-O-glucoside (K2 and K1, respectively; Fig. S3).

**Fig. 4:**
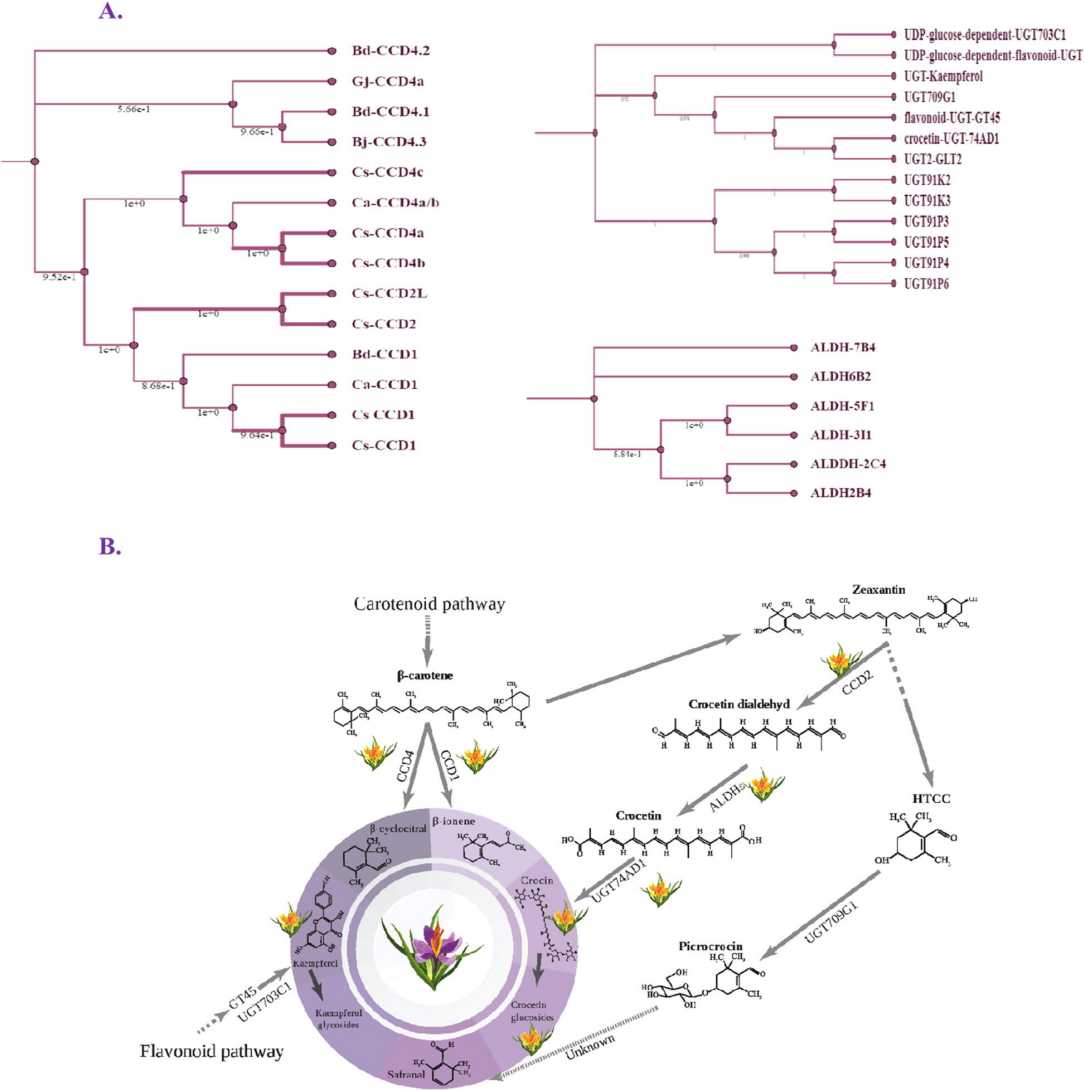
General legend for Figure. **A** Phylogenetic trees of *C. sativus* gene families involved in the biosynthesis of value-added compounds. **B** Major value-added metabolites of *(C) sativus* stigma are shown in the circle along with their pathways and the related genes and metabolites. In this study, the metabolites of the tepals of *C. korolkowii*, *C. graveolens*, *C. flavus* ssp. *dissectus* and *C. chrysanthus* were investigated to check if they are similar to the ones in saffron stigma. The yellow flowers in the figure indicate the metabolites or genes that were detected in the yellow-tepals crocuses

 To gain insights into the biosynthetic genes related to saffron-like metabolites in the four yellow-tepal *Crocus* species, the tepals of *C. chrysanthus*, *C. flavus* subsp. *dissectus*, *C. graveolens*, *C. korolkowii* were used for transcriptomic analysis. A total of 177,738,884 clean reads were obtained across all the samples. We compared the assembled sequences to known *C. sativus* genes involved in apocarotenoid and flavonoid metabolism (Table 1). The candidate genes were categorized into three major groups involved saffron apocarotenoid and flavonoid metabolism: carotenoid cleavage dioxygenases (CCDs), uridine diphosphate glucosyltransferases (UGTs), and aldehyde dehydrogenases (ALDHs). A CCD, CCD2, catalyzes the first step of apocarotenoid biosynthesis in saffron, being responsible for the oxidative cleavage of zeaxanthin and the production of HTTC and crocetin dialdehyde [2]. ALDHs are responsible for the transformation of crocetin dialdehyde into crocetin. The UGTs are responsible for glycosylation of low molecular weight substrates such as crocetin, HTTC, and flavonoid aglycones to produce crocin, picrocrocin, and kaempferol derivatives, respectively. The phylogenetic trees of these gene families are provided, indicating their roles in saffron metabolites pathways (Fig. 4A). Genes associated with the CCD family were selected from both *C. sativus* and other close-related crocus species to encompass a wider range of genetic diversity, as CCD2 is the one characterized/known in *C. sativus*. Conversely, the selection of genes linked to the UGTs and ALDHs families was limited to those exclusively found in *C. sativus*, given the substantial abundance of genes from these families within this particular species.

**Table 1:** Genes related to apocarotenoid and kaempferol biosynthesis pathways of C. sativus in yellow-tepal crocuses

Name	GenBank accession no.	C. korolkowii	C. chrysantus	C. graveolens	C. flavus ssp. dissectus
		E-value/%identitiy	E-value/identitiy%	E-value/identitiy%	E-value/identitiy%
*C. sativus* carotenoid cleavage dioxygenase 2 (CCD2)	KJ541749	1e-161 / 70	0.0 / 73	3.00e-116 / 77	1e-46 / 74
*C. sativus* carotenoid cleavage dioxygenase 2 l (CCD2L)	KP887110	1e-161 / 66	0.0 / 73	1.00e-115 / 77	2e-46 / 74
*C. sativus* chromoplast carotenoid cleavage dioxygenase 4b (CCD4b)	EU523663.1	No hits found	2e-142/87%	0.0/87%	No hits found
*C. sativus* chromoplast carotenoid cleavage dioxygenase 4a (CCD4a)	EU523662.1	No hits found	2e-142/87%	0.0/87%	No hits found
*C. sativus* carotenoid cleavage dioxygenase 1	OL606625.1	0.0/97%	0.0/96%	0.0/96%	3E-139/97%
*C. ancyrensis* carotenoid cleavage dioxygenase (CCD4c)	KP792758.1	0.0/89%	0.0/96%	No hits found	3E-115/90%
*C. ancyrensis* carotenoid cleavage dioxygenase (CCD4a/b)	KP792757.1	4E-133/98%	0.0/99%	0.0/90%	8E-087/89%
*C. ancyrensis* carotenoid cleavage dioxygenase (CCD1)	KP792755.1	0.0/96%	0.0/98%	0.0/98%	9E-146/99%
*C. sativus* carotenoid cleavage dioxygenase 1 (CCD1)	MN540633.1	0.0/97%	0.0/96%	0.0/96%	3E-139/97%
*C. sativus* chromoplast carotenoid cleavage dioxygenase 4b (CCD4b)	EU523663.1	No hits found	2e-142/87%	0.0/87%	No hits found
*C. sativus* UGT709G1 mRNA, complete cds (uridine diphosphate glycosyltransferase)	KX385186.1	3e-62 / 67	0.0 / 61	3e-101 / 38	6e-06 / 40
*C. sativus* glucosyltransferase 2 (GLT2) mRNA	AY262037.1	6e-65 / 74	1e-159 / 61	2.00e-49 / 30	7e-46 / 66
*C. sativus* crocetin glucosyltransferase 74AD1 mRNA	MF596166.1	3e-74 / 36	0.0 / 81	2.00e-56 / 31	2e-51 / 71
*C. sativus* beta-carotene hydroxylase to a 915-base sequence of most likely codons	CAC95130	1e-144 / 87	3e-144 / 86	2.00e-13 / 90	0.62 / 50
*C. sativus* aldehyde dehydrogenase 2B4 (ALDH2B4) mRNA	MG672523.1	0.0 / 98	0.0 / 44	1.00e-125 / 96	2.7 / 47
*C. sativus* aldehyde dehydrogenase ALDH2C4 mRNA, complete	MF596160.1	0.0 / 81	0.0 / 97	2.00e-104 / 82	0.024 / 39
*C. sativus* aldehyde dehydrogenase 3I1 mRNA, complete	MF596165.1	0.0 / 96	4e-102 / 58	1.00e-50 / 94	5.8 / 47
*C. sativus* aldehyde dehydrogenase ALDH5F1 mRNA	MF596161.1	0.0 / 96	4e-102 / 38	1.00e-50 / 94	5.8 / 47
*C. sativus* aldehyde dehydrogenase 6B2 (ALDH6B2) mRNA	MG672524.1	0.0 / 97	0.0 / 97	3.00e-93 / 97	1.4 / 35
*C. sativus* Aldehyde dehydrogenase ALDH7B4 mRNA, complete	MF596162.1	0.0 / 99	0.0 / 99	4.00e-171 / 99	2.3 / 41
*C. sativus* Glucosyltransferase (UGT91P3)	MZ190170.1	4e-90 / 62	7e-121 / 86	2.00e-43 / 92	0.68 / 30
*C. sativus* glucosyltransferase (UGT91P6)	MZ190175.1	4e-117 / 79	1e-100 / 45	6.00e-38 / 31	0.010 / 42
*C. sativus* glucosyltransferase (UGT91K3)	MZ190174.1	1e-69 / 72	0.0 / 43	7.00e-40 / 30	1e-07 / 45
*C. sativus* glucosyltransferase (UGT91K2)	MZ190173.1	5e-67 / 73	0.0 / 78	1.00e-36 / 31	2e-08 / 47
*C. sativus* glucosyltransferase (UGT91P5)	MZ190172.1	2e-97 / 64	1e-121 / 82	1.00e-43 / 92	5.0 / 33
glucosyltransferase (UGT91P4)	MZ190171.1	2e-134 / 81	1e-106 / 46	3.00e-39 / 31	0.011 / 45
*C. sativus* glucosyltransferase (Kaempferol)	HE793682.1	0.0 / 92	0.0 / 90	5.00e-104 / 91	2e-05 / 29
*C. sativus* flavonoid glucosyltransferase (GT45) gene	FJ194947.1	0.0 / 69	0.0 / 70	2.00e-47 / 28	8e-36 / 81
*C. sativus* UDP-glucose-dependent flavonoid UGT703B1	KJ381079	7e-92 / 41	0.0 / 89	0.0 / 68	1e-10 / 34

While only a few of these genes were annotated in *C. flavus* subsp. *dissectus* with a low identity percentage, meaningful values for all the candidate genes were observed in *C. chrysanthus*, *C. graveolens*, and *C. korolkowii* (Table 1). Transcripts of these three species showed a considerable similarity to the candidate saffron genes. When considering the identity percentage of above 70%, the highest number of reads with the highest identity percentage were observed in *C. korolkowii.* In fact, the transcripts of *C. korolkowii* were easily aligned to the main genes involved in the apocarotenoid and flavonoid pathways, including CCD2, UGTs and ALDHs.

Among the assembled transcripts, we observed potential isoforms of some candidate genes including a flavonoid glucosyltransferase (GT45), UDP-glucose-dependent flavonoid glucosyltransferase (UGT703B1), carotenoid cleavage dioxygenase 2 (CCD2), and beta-carotene hydroxylase in *C. chrysanthus*, glucosyltransferase 2 (GLT2) in *C. chrysanthus, C. graveolens* and *C. korolkowii*, aldehyde dehydrogenase 2B4 (ALDH2B4) and glucosyltransferase (kaempferol) in *C. chrysanthus* and *C. korolkowii* (Table S2).

## Discussion

As more research is conducted on the potential health benefits of saffron value-added metabolites, the demand for these compounds is likely to increase. Finding alternative sources of these metabolites could help ensure a stable supply for the market. While historically the stigmas of some other crocuses like *C. cartwrightianus*, the known ancestor of saffron [31, 32], were used as wild saffron, only *C. sativus* stigmas contain a considerable amount of these metabolites. Since previous investigations confirmed the presence of value-added metabolites in saffron tepals, tepals of other crocuses are a potential source worth exploring. In the present work, using the targeted metabolomics and transcriptomics approaches, four yellow-tepal crocus species were investigated: *C. flavus* subsp. *dissectus*, *C. graveolens*, *C. korolkowii* and *C. chrysanthus*.

By examining the saffron apocarotenoid pathway, we could gain a deeper understanding of the genes involved in producing saffron-like metabolites. The presence of various types of crocins was proved in all four species. However, major crocins of *C. sativus* (*t*-C-2, *t-*C-3, *t-*C-4) were only detected in *C. chrysanthus* and *C. korolkowii* (Fig. 3), which is also supported by our transcriptom analysis (Table 1). We analyzed three key enzymes of the apocarotenoid pathway, CCDs, ALDHs, and UGTs. We observed that CCD2, the enzyme catalyzing the first committed step of crocin biosynthesis, is present in the tepals from all of the four yellow-tepal crocuses. We also identified transcripts for several ALDH homologs including ALDH2B4, ALDH2C4, ALDH3I1, ALDH5F1, ALDH6B2, ALDH7B4 (Fig. 4A; Table 1).

The presence of CCD2, ALDH and crocin-related UGTs transcripts was confirmed in tepals of *C. flavus* subsp. *dissectus* and *C. graveolens*, despite no saffron-like crocins being detected in their LC-PDA analysis.

The absence of detected stigma-like crocins in LC-PDA analysis prompted us to explore a combination of three different hypotheses to understand the underlying reason. The post-harvest degradation of crocins could be considered as the first explanation for the absence of crocins. Since all transcripts of the crocin pathway genes were observed in *C. graveolens*, crocins might have become degraded subsequently. The effect of environmental factors on crocins were investigated by HPLC-DAD-MS [33] where light and temperature were introduced as the main elements that could degrade crocins after only one week. Due to the detection of other types of crocins in all four species, as well as their detection in saffron stigmas, this hypothesis is not likely.

We considered the possibility that other compounds and CCD enzymes, rather than those directly involved in the synthesis of saffron-like crocins, may play a more important role in yellow tepal color. Besides crocins, there are various apocarotenoids such as β-cyclocytral and β-ionone which are CCD cleavage products that provide specific aromas and colors ranging from yellow to red in fruits and flowers [34]. CCD2 belongs to the larger family of CCD enzymes which also includes CCD1, CCD4, CCD7, and CCD8 [35]. As it is shown in our phylogenetic analysis (Fig. 4A), candidate genes for CsCCD and CCDs of other crocin-producing plants clustered separately while BdCCD1 clustered in CsCCD. CsCCDs clustered in two groups led by CCD1 and CCD4. The role of CCD1 and CCD4 in providing unique aromas and colors through carotenoid degradation was demonstrated in different plants [36]. For instance, in *Medicago truncatula* CCD1 is responsible for yellow color [37, 38], while in petals of chrysanthemum, CmCCD4a results in colorless compounds in white flowers [39]. In contrast, the reddish color in citrus fruits is attributed to CitCCD4 [12]. Moreover, previous studies validated the presence of CCD1a-, CCD1b- and CCD4a/b-encoding genes in the stigmas of *C. sativus* and in tepals of *C. chrysanthus* and *C. korolkowii* [40]. CsCCD1 and CsCCD4 convert β-carotene into β-ionone and β-cyclocitral [11] that play significant roles in the synthesis of aroma and flavor.

In our study, CCD1 and CCD4 transcripts were also observed in all the studied yellow-tepal species. The presence of these transcripts could contribute to their yellow color along with the presence of crocins (Fig. 4). What is evident is that crocins, although present, are found in low abundance on a dry weight basis compared to saffron stigmas. However, it is essential to consider them as potential commercial sources due to the petals’ significantly higher mass compared to saffron stigmas.

The third and the most probable hypothesis refers to the glycosylation of plant secondary metabolites that is done by UGTs displaying the well-known PSPG (plant secondary-product-glycosyltransferase) box [14]. While more than a hundred UGT-encoding genes exist in each plant genome [41], the saffron-related ones were identified recently [2] that are shown in phylogenetic tree (Fig. 4A). While UGT74AD1 (also named GL2) allows the formation of crocins with one and two glucose molecules, UGT91P3 does not use crocetin as a substrate and generate crocins with more than two glucose molecules [13]. Studies demonstrated the expression patterns of the six identified genes (UGT91K2, UGT91K3, UGT91P3, UGT91P4, UGT91P5 and UGT91P6) of the UGT91 subfamily in several tissues of *C. sativus* [33]. While only UGT91P3 showed higher expression levels in the stigma, higher expression levels of UGT91K3 were detected in leaves [13], suggesting the involvement of the latter in the synthesis of other compounds rather than crocins, which are absent in the leaves. As it is shown in the phylogenetic tree, these genes are grouped together (Fig. 4A). UGT91K2, UGT91K3 and UGT91P3 had expression patterns similar to UGT74AD1 [13]. Based on the Blast analysis of the crocin-related UGTs, we observed a higher identity percentage among UGT74AD1 (GL2), UGT91K2, UGT91K3, UGT91P3, UGT91P4, UGT91P5 and UGT91P in *C. korolkowii* (Table 1). It could potentially confirm the observed variety of crocins at the metabolome level. In contrast, a lower number of these genes with a high identity percentage were found in *C. chrysanthus* and the decreasing trend continued in *C. graveolens* and reached to almost non-significant ones in *C. flavus* subsp. *dissectus*. This observation could also suggest that these species are increasingly distantly related to saffron.

Our metabolome results provide evidence explaining why we still find crocins in yellow tepals, but these differ significantly from those found in the stigmas. The main reason behind these differences could be attributed to variations in the substrate affinity and catalytic properties of the putative crocetin UGTs. Notably, all the detected crocins in the tepals exhibited higher polarity compared to those present in saffron stigmas, leading to their earlier appearance in the LC chromatogram. This higher polarity suggests a greater degree of glycosylation. Investigating glucose availability in the tissues, as glucose is used for glycosylating the crocetin backbone, could be a compelling aspect to explore further. This might shed light on the discrepancy between tepals and stigmas in terms of crocin composition and abundance. This is in line with another study on some yellow-tepal crocuses showing that the crocins in tepals possess higher glycosylation levels in comparison to the crocins in *C. sativus* stigmas [6]. While crocins in saffron stigmas contain up to five and six glucose molecules [42, 43], crocins in yellow-tepal crocuses include up to eight sugar molecules [6]. In fact, these highly glycosylated crocins demonstrate differences in retention times indicating different arrangements of the glucose molecules on the ends of the crocetin [44]. Moreover, previous studies showed that these crocins are also present in the stigmas of spring crocuses and because of their absence in the stigma of autumn crocuses could be considered a distinguishing factor between spring and autumn crocuses [45].

The apocarotenoid HTCC is also glycosylated by UGTs to produce picrocrocin, the precursor of safranal. UGT709G1 catalyzes the HTCC glucosyltransferase reaction [2] (Fig. 4). Neither HTCC, picrocrocin nor safranal were detected in yellow-tepals samples (Fig. 3). Nevertheless, at the transcriptome level, the UGT709G1 was identified in almost all samples. While the final enzyme in safranal production is still unknown [46, 47], we could only check HTCC glucosyltransferase that eventually produces picrocrocin. Considering the unknown safranal pathway, several possibilities could be used to explain the absence of safranal despite the presence of UGT709G1. Furthermore, it has been reported that the presence of picrocrocin and safranal in stigmas is greatly influenced by the developmental stage of the flowers [17]. Conducting a time-course analysis of tepals could potentially shed light on the ability of crocus tepals to produce these monoterpenoids. Alternatively, the substrates of UGT709G1 might differ in a species- and/or tissue-specific manner, leading to variations in the production of certain compounds.

To provide a comprehensive view of the presence of saffron value-added metabolites in yellow-tepal crocuses, the presence of saffron stigma-like kaempferols was also investigated. Our results confirmed the presence of two kaempferol derivatives in *C. korolkowii* that werealso identified in *C. sativus* stigmas. Alongside with kaempferol glycosides, which are the sole flavonoids in saffron stigmas, a higher diversity of flavonoids, such as anthocyanins, quercetin- and flavone-related compounds (luteolin, tricin, acacetin, apigenin, and scutellarein) are found in saffron tepals [48, 49]. The detection of multiple compounds with maximum UV-Vis absorption values at 320 nm in all tepal samples, supports the presence of a higher diversity of flavonoids in the yellow-tepal crocuses. Analysis of *C. chrysanthus* tepals has proved the existence of kaempferol *3-O* glucoside [50]. Other flavonoids such as luteolin-derivatives in *C. corsicus* and *C. minimus*, and tricin in *C. heuffelianus* and *C. korolkowii* have been reported [51]. In our study, we found flavonoid glucosyltransferase transcripts with the highest to the lowest E-value and identity percentage in *C. korolkowii*, *C. chrysanthus*, *C. graveolens* and *C. flavus* subsp. *dissectus*, respectively.

Ultimately, tepals of *C. korolkowii* with three proven types of saffron-like crocins and two kaempferol-derivatives showed the highest similarity to saffron stigmas. It is important to highlight that additional crocin species were found in yellow tepals which despite differing from the ones in saffron, support the metabolic capability of these tissues and species to synthesize crocins. The identified crocins differ in the decorations of the crocetin backbone, likely displaying high glycosylation levels. Although the crocins found in the tepals are different from those in saffron stigmas, they could still be a potential source of pigments that meet industrial demands. The identification of additional CCD transcripts in the yellow crocuses may also be involved in the synthesis of non-crocin pigments, which could also be of commercial interest. Moreover, our results support the presence of diverse flavonoid in tepals, which could be exploited as a source of natural products for nutritional and medicinal purposes.

This study could be considered as the primary objective to uncover insights into the biosynthesis of compounds within saffron stigmas; subsequently, determining the presence or absence of saffron stigma-like metabolites in yellow-tepals crocuses. Further investigations like gene expression analysis could be beneficial for future goals when the applications of various crocins could get more attention.

## Conclusions

Previous observations noted that saffron tepals contain metabolites similar to the ones in stigma, leading to recent exploration of tepals as a potential compound source. Our study focused on the metabolomic and transcriptomic analysis of saffron stigma-like apocarotenoids and kaempferol-derivatives in the tepals of four common yellow-tepal crocuses.

Among these crocuses, the metabolites in *C. korolkowii* tepals displayed the highest similarity to those in saffron stigmas, containing three verified stigma-like crocins and two kaempferol-derivatives. However, most crocins in yellow tepals differed structurally from those in saffron, featuring variations in crocetin backbone decorations and increased glycosylation. Despite these distinctions, the identified crocins in tepals remain a potential source for industrial pigments.

Moreover, our results indicated a diverse array of flavonoids in tepals, presenting an additional opportunity for exploration. These flavonoids hold promise as a source for the development of natural products with applications in nutrition and medicine. In summary, while crocins in yellow-tepal crocus tepals differ from those in saffron stigmas, they still hold potential for industrial pigments. Additionally, the presence of diverse flavonoids in tepals expands the potential applications of these crocuses, offering opportunities for the development of natural products with nutritional and medicinal benefits.

## Methods

### Plant materials

Stigmas of the autumn-flowering saffron crocus (*C. sativus*) along with tepal samples of four species each of the yellow, spring-flowering species *C. flavus* subsp. *dissectus*, *C. graveolens* and *C. korolkowii* were grown and collected in IPK Gatersleben greenhouses while *C. chrysanthus* was provided by Vladimir Randjelovic from the University of Niš. Samples for metabolite and transcriptome analyses were collected with two different methods. For metabolic analysis, tepals and stigmas were fast-dried in silica gel and stored dry till their analysis. For the transcriptome analyses, tissue samples of tepals and stigmas were stabilized in RNA*later* (Qiagen, Germany) to preserve RNA integrity during storage.

### Metabolite extraction and analysis

Soluble semi-polar metabolites were extracted from tepal and stigma samples. All extraction steps were performed at room temperature under darkness. Firstly, samples were transferred into 2-ml Eppendorf tubes and weighted (5 to 15 mg). These were added with 100 µl of 50% (v/v) methanol per mg of material. Samples were grinded using a Precellys homogeneizer (Bertin Instruments, France) using 1–1.2 mm-diameter zirconium silicate beads (Mühlmeier GmbH, Germany) with four cycles of 20 s each, at a frequency of 6000 Hz. Samples were vortexed and extracted through continuous agitation at 500 rpm and room temperature for 20 min under dark conditions (Eppendorf ThermoMixer, Germany). Samples were centrifuged at 17,000 g for 5 min and the supernatant was transferred into a new tube. The pellet was re-extracted two more times, each by adding 100 µl of 50% (v/v) methanol per mg of material, followed by vortexing, shaking (20 min, 500 rpm), and centrifugation. In each step, the supernatants were combined with the first one. Extracts were covered with Argon and stored at -20 °C prior to analysis.

Semi-polar metabolites were analyzed by reversed phase ultra-performance liquid chromatography coupled with photodiode array detection (RP-UPLC-PDA), using an Acquity UPLC system (Waters, Germany) equipped with an Acquity UPLC PDA eλ detector (Waters, Germany). Extracts were first centrifuged at 17,000 *g* for 10 min, and 100 µl of supernatant were transferred into glass vials for analysis. Sample injection volumes of 5 µl were employed for UPLC analysis, using a partial-loop with needle-overfill (PLNO) injection mode with a 10 µl loop. Compounds were separated in an Acquity UPLC BEH phenyl column (130 Å, 2.1 × 100 mm, 1.7 μm; Waters, Germany) combined with an Acquity UPLC BEH phenyl VanGuard pre-column (130 Å, 2.1 × 5 mm, 1.7 μm; Waters, Germany) using the following gradient: from 10 to 36% of solvent B for the first 3.9 min, isocratic hold from 3.9 to 5 min, from 36 to 97% B from 5 to 6 min, and an isocratic hold from 6 to 7 min to clean the column; after each run, the column was equilibrated to the starting conditions (10% B); solvent A was LC-MS grade water (CHEMSOLUTE, Th. Geyer, Germany) with 0.5% (v/v) formic acid, and solvent B was LC-MS grade acetonitrile (CHEMSOLUTE, Th. Geyer, Germany) with 0.5% (v/v) formic acid. The column temperature was maintained at 35 °C and the flow was set to 500 µl min^-1^. PDA-detection was performed in a range between 210 and 800 nm, at a resolution of 1.2 nm and a sampling rate of 20 points s^-1^. Within the major *Crocus* semi-polar metabolites, crocins, picrocrocin, and flavonoids and safranal, were detected at 440, 250, and 320 nm, respectively. The identity of crocin (*trans*-crocetin-digentibiosyl ester), picrocrocin, and safranal, was confirmed by retention time and UV absorption spectra with commercial standards. Processing and analysis of the acquired PDA spectra was done with the Empower 3 software (Waters, Germany).

To generate a reliable reference composition, the extracts from *C. sativus* stigmas were further analyzed via ESI-UHR-QTOF-MS (ElectroSpray Ionization-Ultra-High-Resolution-Quadrupole Time of Flight-Mass Spectrometry) by coupling a maXis Impact ESI-QTOF MS (Bruker Daltonik GmbH; Germany) to the RP-UPLC-PDA system. The MS analyses were performed in positive and negative ionization modes. Small molecules (i.e., safranal, picrocrocin, flavonoids; 50-1000 *m/z*) were analyzed using the MS1 and MS/MS settings for barley and sunflower phenylpropanoids described in [52]. Large molecules (i.e., crocins; 50-1500 *m/z*) were analyzed with the MS1 settings in positive ionization mode for anthocyanins as described in [52]. The MS/MS analysis of large molecules in positive mode was performed in auto MS/MS using CID (Collision-Induced Dissociation) with the following settings: absolute area threshold: 5000 counts; exclusion activation: 15 spectra; exclusion release: 30 s; collision energy values (z = 1, 2, 3; isolation mass = 500; width = 8): 35, 25, 20 eV; collision energy values (z = 1, 2, 3; isolation mass = 1000; width = 10): 50, 40, 35 eV. The analysis of large molecules in negative mode was done with the following MS1 settings: 50–1000 *m/z*; capillary voltage: 3.5 kV; nebulizer: 3 bar; dry gas: 8 l min^−1^; dry temperature: 200 °C; hexapole RF (Ratio Frequency) voltage: 150 Vpp (V peak-to-peak); funnel 1 RF: 400 Vpp; funnel 2 RF: 400 Vpp; pre-pulse storage time: 8 µs; transfer time: 60 µs; low mass: 40 *m/z*; collision cell RF: 800 Vpp; collision energy: 8 eV. The MS/MS analysis of large molecules in negative mode was done in auto MS/MS using CID as follows: absolute area threshold: 5000 counts; exclusion activation: 2 spectra; exclusion release: 12 s; collision energy values (z = 1; isolation mass = 500; width = 6): 20 eV; collision energy values (z = 1; isolation mass = 1000; width = 8): 20 eV. The Compass HyStar 3.2 SR2 software (Bruker Daltonik GmbH, Germany) was used to operate and coordinate LC-PDA-MS data acquisition. The analysis of MS data was performed using the Compass DataAnalysis 4.4 SR1 package (Bruker Daltonik GmbH).

### RNA extraction and transcriptome sequencing

Total RNA was extracted using the Qiagen RNeasy plant mini kit. Total RNA purity and concentration were determined by the Qubit 1 2.0 Flurometer (Life Technologies, USA). RNA integrity was assessed using the RNA Nano 6000 Assay Kit of the Agilent Bioanalyzer 2100 system (Agilent Technologies, USA). A minimum amount of 50 ng µl^-1^ RNA per sample was used as input material for the RNA sample preparations. Sequencing libraries were generated using the llumina TruSeq RNA Sample Prep Kit v2 for Illumina sequencing platforms.

### Transcriptome assembly and annotation

The library preparations were sequenced on an Illumina Novaseq 6000 platform and paired-end reads were generated. In the quality control step, raw reads of fastq format were first processed through trimmomatic [53]. All the downstream analyses were based on clean data with high quality. The transcriptome was assembled using Trinity-v2.9.0 [54] with min_kmer_cov set to 2 by default and all other parameters set default.

Genes linked to crocin and kaempferol biosynthesis in *C. sativus* were selected from the literature [7, 13, 20, 55–57]. These genes are listed in Table 1 and were subsequently used as a database in TBlastN [58]and potential orthologs of these genes in the Trinity transcriptome assemblies were searched using the TBlastN parameters with e-value cut-of 0.01. Genes were then selected as potential full-length orthologs if they contained an ORF that had an overlap with at least 90% of the reference gene. Shorter sequences were listed as partial genes (Table S2).

Phylogenetic trees for three main genes (CCDs, ALDHs and UGTs) are calculated using MEGA3 [59] with ClustalW [60] alignment along with maximum likelihood with bootstrap 500 to generate the trees.

### Supplementary Information

**Additional file 1:** **Fig. S1.** Representative crocins present in tissues from *Crocus *species. Representative UV-Vis absorption spectra for major crocins detected in the analyzed samples. **Fig. S2.** Major crocins in *C. sativus* stigmas and yellow tepals from multiple *Crocus* species. **Fig. S3.** Analysis of flavonoids and safranal in tissues from multiple *Crocus* species. **Fig. S4.** Analysis of HTTC and picrocrocin in tissues from multiple *Crocus *species. **Table S1.** Table of metabolites quantities detected in *C. sativus* and yellow-tepal crocuses. **Table S2.** List of all potential isoform genes resulted by TBlastN.

## Data Availability

The RNA-seq data for this study can be accessed at the NCBI Sequence Read Archive (http://www.ncbi.nlm.nih.gov/sra) under accession number PRJNA926329.

## Abbreviations

ALDH: Aldehyde dehydrogenase
CHY-β: β-Carotene hydrolase
BLAST: Basic Local Alignment Search Tool
CCD: Carotenoid Cleavage Dioxygenase
c-C: Cis-crocetin
ESI-UHR-QTOF-MS: ElectroSpray Ionization-Ultra-High-Resolution-Quadrupole Time of Flight-Mass Spectrometry
GT: Glycosyltransferase
HPLC-DAD-MS: High-Performance Liquid Chromatography coupled to Diode Array Detection and Mass Spectrometry
HTCC: 2,6,6-Trimethyl-4-hydroxy-1-carboxyaldehyde-1-cyclohexene
K1: Kaempferol 3-O-sophoroside 7-O-glucoside
K2: Kaempferol 3-O-sophoroside
LC: Liquid Chromatography
LC-PDA: Liquid Chromatography coupled to Photodiode Array
LCYB: Lycopene β-cyclase
LC-MS: Liquid Chromatography-Mass Spectrometry
MEP: Methylerythritol phosphate
MS: Mass Spectrometry
MVA: Mevalonate pathways
ORF: Open Reading Frame
PDA: Photodiode Array Detection
PSY: Phytoene synthase
PSPG: Plant Secondary-Product-Glycosyltransferase
QTOF: Quadrupole Time of Flight
RNA: Ribonucleic Acid
RP-UPLC-PDA: Reversed Phase Ultra-Performance Liquid Chromatography coupled with Photodiode Array Detection
t-C: Trans-crocetin
TBLASTN: Translated Basic Local Alignment Search Tool with Nucleotides
UGTs: Uridine Diphosphate Glucosyltransferases
