# Supplementary material for: Metabolomic and transcriptomic analyses of yellow-flowered crocuses to infer alternative sources of saffron metabolites
Source: BMC Plant Biol. 2024 May 7;24:369. doi: 10.1186/s12870-024-05036-1 (PMC11075242; doi:10.1186/s12870-024-05036-1)
Supplement: Supplementary file 1 — Additional file 1: Fig. S1. Representative crocins present in tissues from Crocus species. Representative UV-Vis absorption spectra for major crocins detected in the analyzed samples. Fig. S2. Major crocins in C. sativus stigmas and yellow tepals from multiple Crocus species. Fig. S3. Analysis of flavonoids and safranal in tissues from multiple Crocus species. Fig. S4. Analysis of HTTC and picrocrocin in tissues from multiple Crocus species. Table S1. Table of metabolites quantities detected in C. sativus and yellow-tepal crocuses. Table S2. List of all potential isoform genes resulted by TBlastN. [file 12870_2024_5036_MOESM1_ESM.docx]

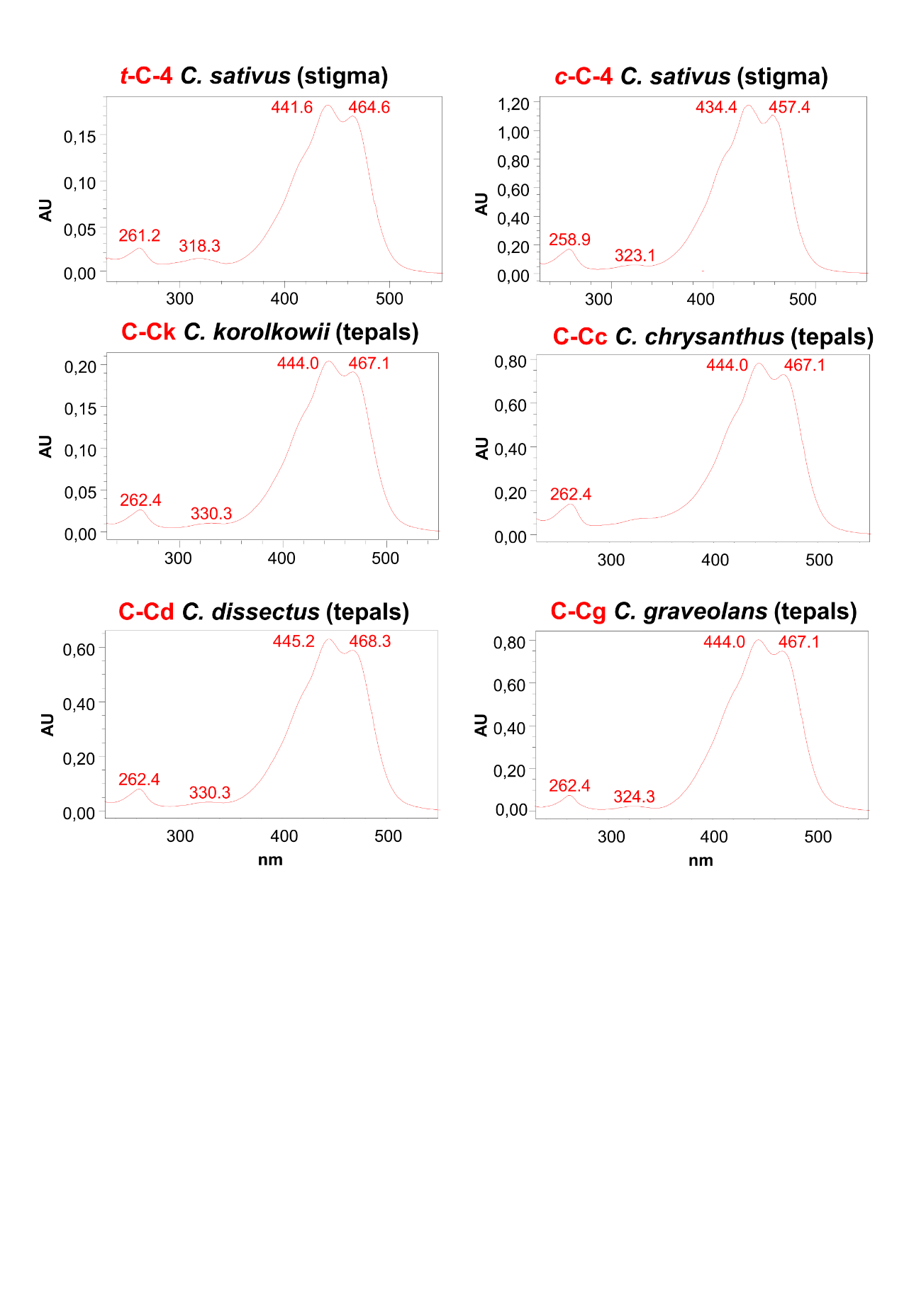


**Fig. S1**. Representative crocins present in tissues from *Crocus* species. Representative UV-Vis absorption spectra for major crocins detected in the analyzed samples. Crocin labels correspond to compounds in Fig. S2 and Table S2.


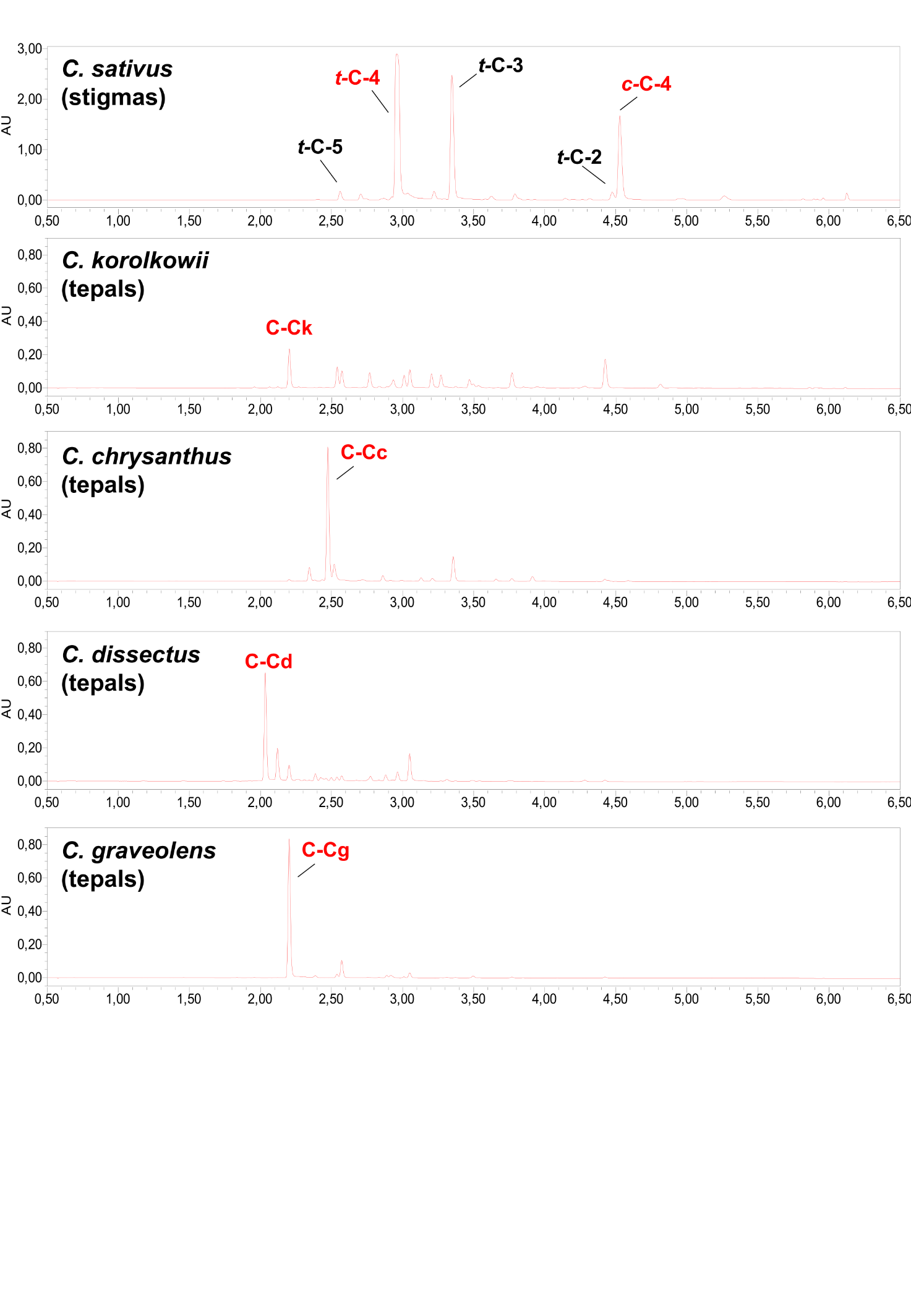
**Fig. S2**. Major crocins in *C. sativus* stigmas and yellow tepals from multiple *Crocus* species. Representative LC-PDA chromatograms extracted at 440 nm. The UV-Vis absorption spectra for major crocins (red labels) are given in Figure5.


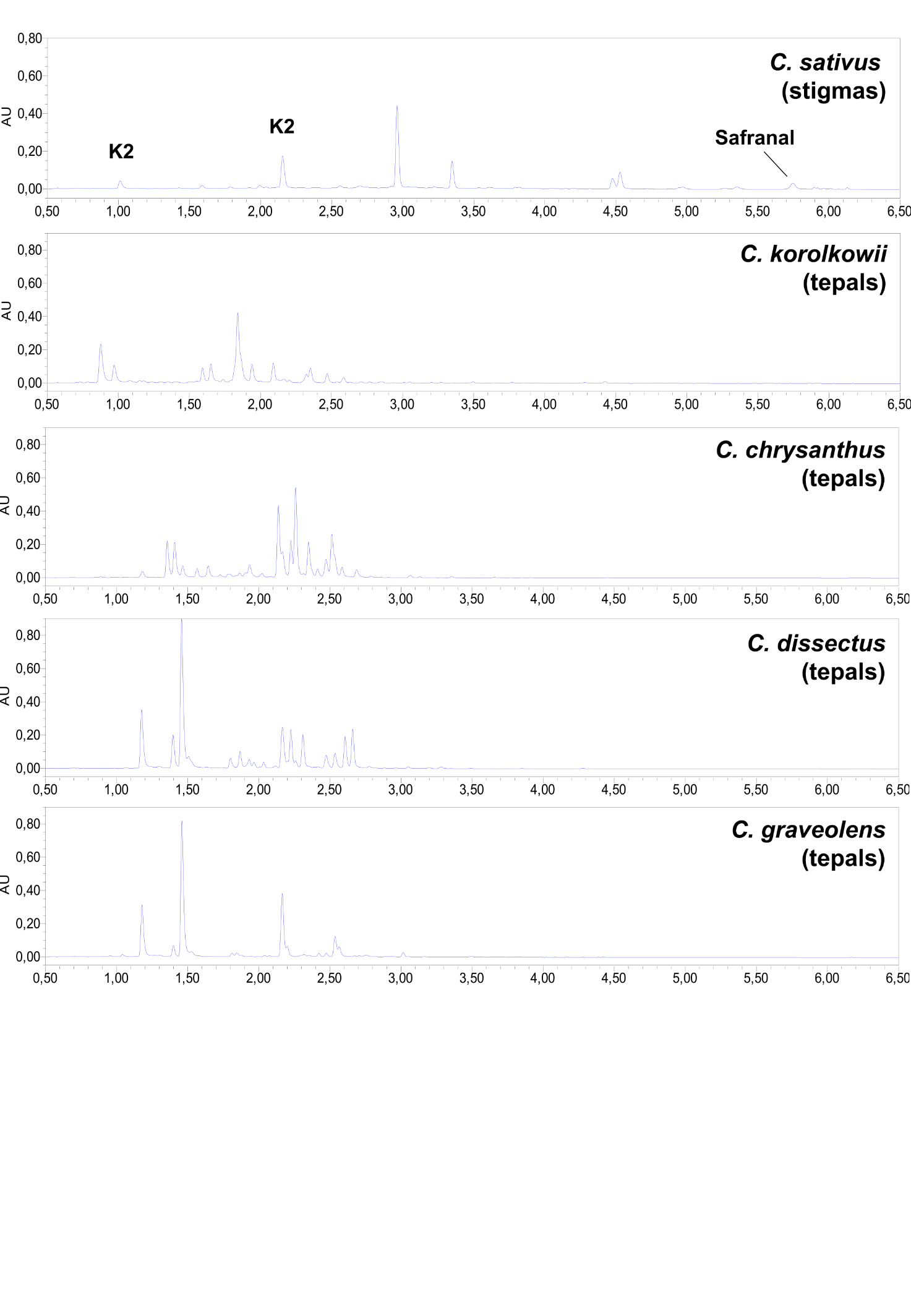
**Fig. S3**. Analysis of flavonoids and safranal in tissues from multiple *Crocus* species. Representative LC-PDA chromatograms extracted at 320 nm.


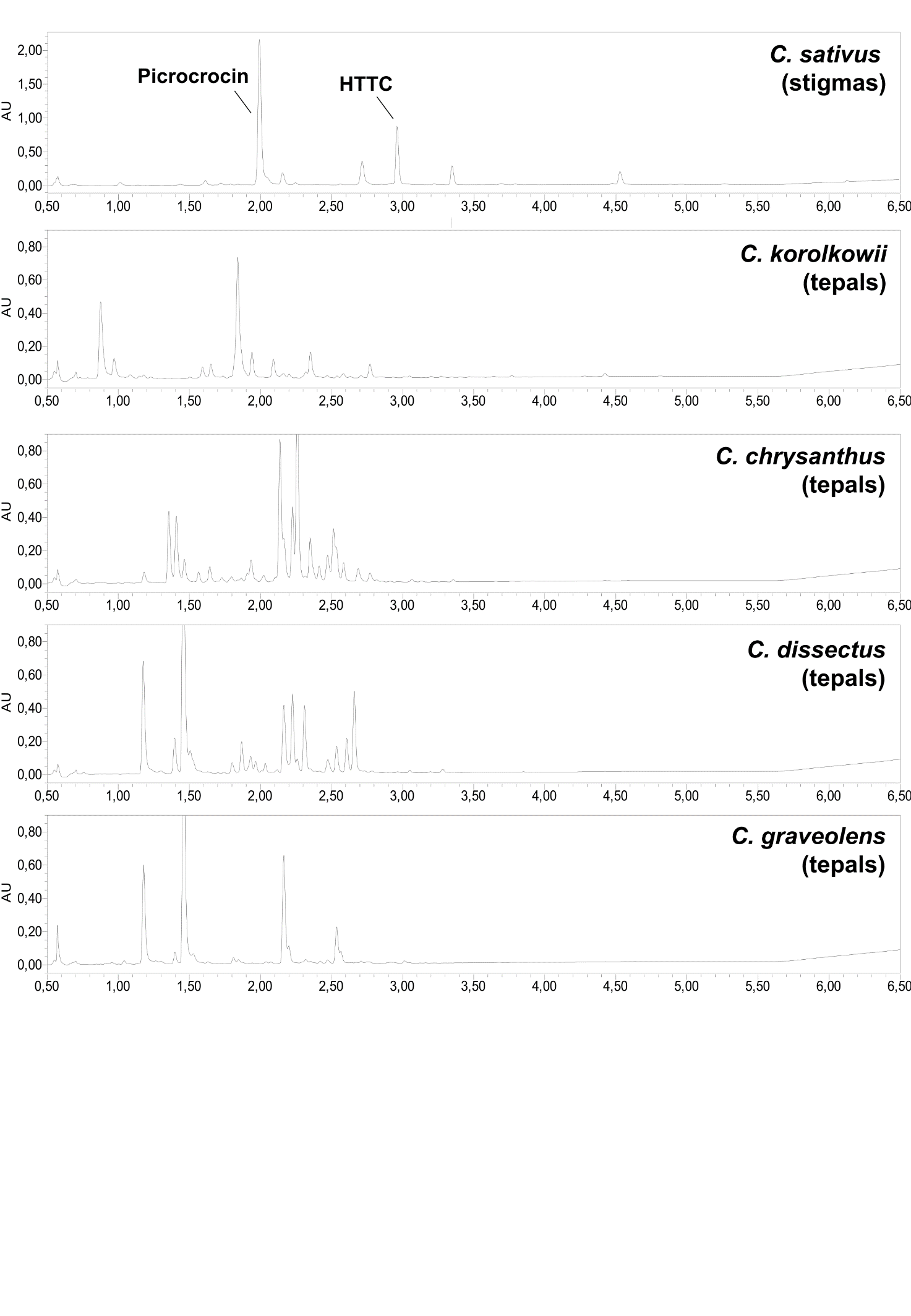


**Fig. S4**. Analysis of HTTC and picrocrocin in tissues from multiple *Crocus* species. Representative LC-PDA chromatograms extracted at 250 nm.

**Table S1.** Table of metabolites quantities detected in *C. sativus* and yellow-tepal crocuses. Units: Absorbance Units (x106) per mg dry weight.

| Species | Flavonoid-K1 | Flavonoid-K4 | Crocint-C-5 | Crocint-C-4 | Crocint-C-3 | Crocint-C-2 | Crocinc-C-4 | Crocinc-C-3 | Monoterpenoid Picrocrocin (P2) | Monoterpenoid HTCC (H) | Monoterpenoid Safranal (S) |
| --- | --- | --- | --- | --- | --- | --- | --- | --- | --- | --- | --- |
| Crocus flavus ssp. Dissectus | 0 | 0 | 0 | 0 | 0 | 0 | 0 | 0 | 0 | 0 | 0 |
| Crocus graveolens | 0 | 0 | 0 | 0 | 0 | 0 | 0 | 0 | 0 | 0 | 0 |
| Crocus chrysantus | 0 | 0 | 5.54 | 0 | 0 | 0 | 0 | 0 | 0 | 0 | 0 |
| Crocus korolkowii | 9.23 | 8.98 | 8.56 | 3.45 | 0 | 14.15 | 0 | 0 | 0 | 0 | 0 |
| Crocus sativus | 3.2 | 8.76 | 49.24 | 778.98 | 253.35 | 56.89 | 321.77 | 30.51 | 110.08 | 61.08 | 10.75 |

**Table S2.** List of all potential isoform genes resulted by TBlastN. The activity and pivotal role of these genes in saffron metabolism have been proved, indicating these isoforms as homologous to the enzymes that have already been characterized.

|  | Trinity sequences | | |
| --- | --- | --- | --- |
| Gene | *C. chrysantus* | *C. korolkowii* | *C. graveolens* |
| FJ194947.1 Crocus sativus flavonoid glucosyltransferase (GT45) gene, complete cds; plastid. | TRINITY_DN17808_c0_g1_i1  TRINITY_DN17808_c0_g1_i2 |  |  |
| KJ381079 Crocus sativus UDP-glucose-dependent flavonoid glucosyltransferase | TRINITY_DN18088_c0_g1_i1  TRINITY_DN18088_c0_g1_i2  TRINITY_DN18224_c0_g1_i1  TRINITY_DN18224_c0_g1_i2 |  |  |
| KJ541749.1 Crocus sativus carotenoid cleavage dioxygenase 2 (CCD2) mRNA, complete cds | TRINITY_DN19662_c0_g1_i1  TRINITY_DN19662_c0_g1_i2 |  |  |
| AY262037.1 Crocus sativus glucosyltransferase 2 (GLT2) mRNA,  complete cds | TRINITY_DN21113_c0_g1_i1  TRINITY_DN21113_c0_g1_i2  TRINITY_DN21422_c0_g1_i1  TRINITY_DN21422_c0_g1_i2  TRINITY_DN21422_c0_g1_i3  TRINITY_DN21602_c0_g1_i1  TRINITY_DN21602_c0_g1_i2  TRINITY_DN22822_c0_g1_i1  TRINITY_DN22822_c0_g1_i3  TRINITY_DN22822_c0_g1_i4  TRINITY_DN22876_c0_g1_i1  TRINITY_DN22876_c0_g1_i2  TRINITY_DN22876_c0_g1_i3  TRINITY_DN23109_c0_g1_i1  TRINITY_DN23109_c0_g1_i2  TRINITY_DN23435_c0_g1_i1  TRINITY_DN23435_c0_g1_i2  TRINITY_DN23435_c0_g1_i3  TRINITY_DN23435_c0_g1_i4  TRINITY_DN23435_c0_g1_i5  TRINITY_DN23435_c0_g1_i6  TRINITY_DN23435_c0_g2_i1  TRINITY_DN23484_c0_g1_i1  TRINITY_DN23484_c0_g1_i2  TRINITY_DN23484_c0_g1_i3  TRINITY_DN23639_c0_g1_i1  TRINITY_DN23639_c0_g1_i2  TRINITY_DN23639_c0_g2_i1  TRINITY_DN23639_c0_g2_i2  TRINITY_DN23722_c0_g1_i1  TRINITY_DN23722_c0_g1_i2  TRINITY_DN23722_c0_g2_i2  TRINITY_DN23722_c0_g2_i3  TRINITY_DN23722_c0_g2_i4  TRINITY_DN23722_c0_g3_i1 | TRINITY_DN18822_c0_g1_i1  TRINITY_DN18822_c0_g1_i2  TRINITY_DN18822_c0_g1_i3  TRINITY_DN18822_c0_g1_i5 | TRINITY_DN7718_c0_g1_i1  TRINITY_DN7718_c0_g1_i2 |
| CAC95130.2 beta-carotene hydroxylase [Crocus sativus] to a 915 base sequence of most likely codons | TRINITY_DN24119_c0_g1_i1  TRINITY_DN24119_c0_g1_i2  TRINITY_DN24119_c0_g1_i3  TRINITY_DN24119_c0_g1_i4  TRINITY_DN24119_c0_g1_i5 |  |  |
| MG672523.1 Crocus sativus aldehyde dehydrogenase 2B4 (ALDH2B4) mRNA, complete cds |  | TRINITY_DN12138_c0_g1_i1  TRINITY_DN12138_c0_g1_i2  TRINITY_DN18615_c0_g1_i1  TRINITY_DN18615_c0_g1_i2  TRINITY_DN18615_c0_g1_i3  TRINITY_DN18615_c0_g1_i4  TRINITY_DN18615_c0_g2_i1 |  |
| UGT707B1 Crocus sativus mRNA for glucosyltransferase (Kaempferol) |  | TRINITY_DN1398_c0_g1_i1  TRINITY_DN1398_c0_g1_i2 |  |
